# Acid-Sensing Ion Channel 2: Function and Modulation

**DOI:** 10.3390/membranes12020113

**Published:** 2022-01-19

**Authors:** Andy Sivils, Felix Yang, John Q. Wang, Xiang-Ping Chu

**Affiliations:** Department of Biomedical Sciences, School of Medicine, University of Missouri, Kansas City, MO 64108, USA; andysivils@umkc.edu (A.S.); felixyang@umkc.edu (F.Y.); WangJQ@umkc.edu (J.Q.W.)

**Keywords:** acid-sensing ion channels, ASIC2, function, physiology, pathology, pharmacology, modulation

## Abstract

Acid-sensing ion channels (ASICs) have an important influence on human physiology and pathology. They are members of the degenerin/epithelial sodium channel family. Four genes encode at least six subunits, which combine to form a variety of homotrimers and heterotrimers. Of these, ASIC1a homotrimers and ASIC1a/2 heterotrimers are most widely expressed in the central nervous system (CNS). Investigations into the function of ASIC1a in the CNS have revealed a wealth of information, culminating in multiple contemporary reviews. The lesser-studied ASIC2 subunits are in need of examination. This review will focus on ASIC2 in health and disease, with discussions of its role in modulating ASIC function, synaptic targeting, cardiovascular responses, and pharmacology, while exploring evidence of its influence in pathologies such as ischemic brain injury, multiple sclerosis, epilepsy, migraines, drug addiction, etc. This information substantiates the ASIC2 protein as a potential therapeutic target for various neurological, psychological, and cerebrovascular diseases.

## 1. Introduction

Neurotransmission is the fundamental process through which neurons store and transfer information. The most well-known subset of this transmissive process is the release of neurotransmitters from presynaptic terminals which then bind to their receptors on postsynaptic membranes, leading to the depolarization of the receiving neuron. For example, glutamate is an excitatory neurotransmitter in the brain, which binds to postsynaptic glutamate receptors after its release, triggers membrane depolarization, or affects intracellular signaling molecules [1,2]. It thus contributes to synaptic plasticity, learning/memory, and plays critical roles in neurological and psychological diseases [1,2]. A lesser-known feature of neurotransmission is the postsynaptic current that only arises from a drop in pH [3]. These currents were observed in dorsal root ganglion (DRG) neurons after a drop in pH from 7.4 to below 7 as early as 1980 by Krishtal’s group [4,5], and have since been investigated to reveal a collection of proteins that facilitate a multitude of neurological and psychological phenomena [6].

Collections of these proteins were cloned by Lazdunski’s group in 1997, and are referred to as acid-sensing ion channels (ASICs) [7]. They are members of the greater degenerin/epithelial sodium ion channel (DEG/ENaC) family. Activation of ASICs largely generates currents via sodium transit across membranes in a voltage-insensitive manner, and has been associated with neurological, psychological, cardiovascular, muscular, skin, and gastrointestinal functions [6,7,8]. There are four different genes (*ACCN1* to *4*) that encode at least six different subunits (ASIC1a, ASIC1b, ASIC2a, ASIC2b, ASIC3, and ASIC4) [6,8]. ASIC1a and ASIC2 are expressed at high levels in the central nervous system (CNS), where they form homotrimeric and heterotrimeric complexes [8,9,10].

**Figure 1:**
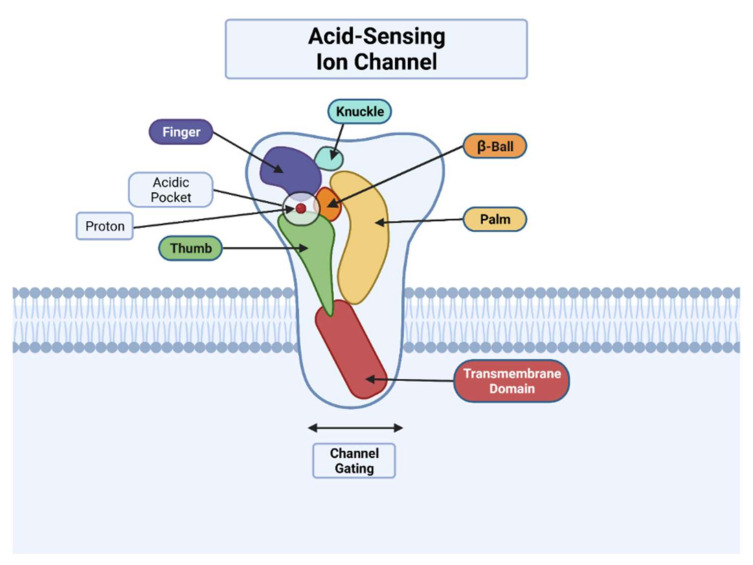
“Closed-fist” model of ASICs. An ASIC subunit has a “closed-fist” morphology, consisting of five protein domains, and each is visualized above. Also depicted is the acidic pocket located between the finger and thumb domains of one ASIC subunit and the palm domain of another subunit. Proton binding to the acidic pocket activates ASICs and results in sodium ion permeation. In addition to the sodium permeability of ASICs, certain ASIC subunits such as homomeric ASIC1a also have calcium permeability. Adapted from “Transporters” by BioRender.com (2021). Retrieved from https://app.biorender.com/biorender-templates, accessed on 13 January 2022.

Consistent with all members of the DEG/ENaC family, individual ASIC subunits comprise more than 500 amino acids and are characterized by intracellular NH_2_ and COOH terminals and two transmembrane domains (TM1 and TM2) [11,12,13,14]. The gross morphology of the ASICs is comparable to a “closed fist”, consisting of a large extracellular conformation made by five protein domains, including the palm, finger, knuckle, thumb, and β-ball domain (Figure 1) [11,12,13]. The palm domain acts as the central structure for each of the other domains, with the β-ball chain located between the palm and finger domains [11]. The “wrist” region, which is closer to the cellular membrane, supports the “hand” region of ASIC domains (palm, finger, knuckle, thumb, and β-ball) [11]. In terms of protein structure for these extracellular domain subunits, they are made up of 12 β-sheets (β1–12), 7 α-helices (α1–7), and 7 disulfide bonds [11,13]. Furthermore, the wrist region connects the palm domain to the two TMs, consisting of six α-helix chains, and these two TMs make up the “pore” region of ASICs that allows sodium and calcium ion permeation [13,14,15]. Ion permeation is triggered by proton binding to the “acidic pocket” of ASIC, which is in the junction between the finger and thumb domain of one ASIC subunit and the palm domain of another subunit [16]. Together, these subunits combine to form homotrimeric or heterotrimeric ion channels that function in channel gating, ion permeation, extracellular and intracellular molecule recognition via ligand binding and protein activation [12,13].

Evidence has shown that ASICs are potential therapeutic targets for conditions such as ischemic brain injury [17], epilepsy [18], migraines [19], intervertebral disc degeneration [20], arthritis [21], addiction [22,23], Parkinson’s disease (PD) [24], Alzheimer’s disease (AD) [25,26], pain [27], and more [28]. However, some of these examinations focused on ASIC1a [21,25]. It is reasonable to think that the ASIC2 variants could be specific therapeutic targets on their own, given that their physiology and pathology have been shown to involve pain modulation [27], mechanosensation [15], acidosis-induced neuronal death [17,28,29], and neurotransmission [22]. Expansion on the physiology, pathology, and modulation of ASIC2 will be informative for the field in assessing its viability as a potential therapeutic target.

Sensitivity to protons in an acidic environment is an integral part of the physiology of ASICs because proton ligands bind to extracellular domains of ASICs and induce channel activation [7,12]. Within areas of high ASIC expression, research has shown that ASICs play a critical role in neuromodulation, related to pH fluctuations in homeostatic metabolism as well as in pathological scenarios with inflammation, hypercapnia, hypoxia, or ischemia [26,28]. The higher expression of proton-gated cation channels in regions of the CNS further supports the idea that ASIC activation due to proton binding is not only a by-product of in vivo acidosis, but an event serving an essential role in communication and neuromodulation in the CNS [7,28].

ASIC2 exhibits a different sensitivity to pH, permeability to ions, and change in response to different subunit compositions as compared with the ASIC1a subunit [11,12,13]. For example, ASIC2a is less sensitive to pH than its ASIC1a counterpart [12]. Additionally, ASIC1a homotrimers and ASIC1a/2b heterotrimers are permeable to both Ca^2+^ and Na^+^, whereas other conglomerates are permeable mainly to Na^+^ [11,12,13]. The ASIC1a, ASIC2a, and ASIC2b subtypes are predominantly found in the brain, with the ASIC1a subtype being the most densely populated [11,12,13]. Investigation into these combinations showed that most functional ASICs in the striatum and cerebellum are ASIC1a homomers [10,15], whereas the majority in all other areas of the brain, such as the hippocampus and amygdala, are homotrimeric ASIC1a and ASIC1a/2 heterotrimers [9,10]. The non-functional ASIC2b subunit itself is expressed not only in the DRG, but also in the brain [29,30]. It associates with other ASIC subunits to form functional channels such as ASIC1a or ASIC3 [29,30]. Although ASIC3 is found primarily in the periphery [31], its brain expression is mostly limited to the mesencephalic trigeminal nucleus; brain ASIC3 expression impairs fear conditioning [28,32]. ASIC4 itself does not form a functional channel, but it is expressed throughout the brain in specific cell types [6,33,34,35]. In the cortex, it is expressed in cortical-calretinin-positive and/or vasoactive-intestinal-polypeptide-positive interneurons and neural/glial antigen 2 polydendrocytes [35]. In the cerebellum, it is expressed in granule cells [35].

To measure and compare the differences in pH sensitivity between ASIC1 and ASIC2, one research group generated an ASIC1a mutant known as “ASIC1a–G430C” due to a G430C cysteine substitution [36]. Similarly, they reproduced the ASIC1a–G430C cysteine substitution at the corresponding position in ASIC2, giving rise to an ASIC2a–A427C mutant [36]. They observed the sustained inward current elicited by different pH levels in both mutants, and found that ASIC2a showed less sensitivity to pH. Sustained inward currents in ASIC2a were only seen with lower pH levels, with and without covalent reagent modifications to their mutant ASIC1a and ASIC2a [36]. ASIC1a demonstrated strong transient inward currents at pH values less than 7.0, whereas ASIC2a WT and ASIC2a mutants were activated at pH values less than 6.0 and 6.5, respectively, lower activation threshold pH values than that of ASIC1a [36]. Another study found that ASIC2a homomers required a pH below 5 to be activated; however, they noted that the in vivo level is likely higher [37]. ASIC2b does not conduct acid-activated inward-currents on its own, but it does affect the expression of ASIC1a [29]. Due to this pH-insensitive property, one might conclude that ASIC2 has a comparatively minimal role in physiological or pathological activities. However, evidence from ongoing research did not support this notion [17,29,38].

There are a couple physiological reasons for ASIC2 pH sensitivity. As previously discussed, one reason is that ASIC2, when associated with other subunits (e.g., ASIC1a or ASIC3), can act as a sensor in acidotic, ischemic environments [38,39,40]. In addition to the ASIC1a, ASIC2a/3 heteromers are seen to have pH-evoked currents in cardiac DRG neurons which function in afferent cardiac signaling [39]. These cardiac afferent neurons primarily act as chemoreceptors that contribute to cardiac reflexes and pain sensations during myocardial ischemia. In the case of ASIC2a/3 heteromers, the threshold of activation is around pH values of 7.4 to 7.2, and this range is shifted to be more sensitive in myocardial infarction, where the main proton carrier in serum is lactate [39,40]. Findings from the middle cerebral artery occlusion (MCAO) and cardiac DRG analysis of ASIC2 in rodents make the channel a possible therapeutic target for reducing both neuronal damage and angina in ischemic-related injuries [17,38,39].

## 2. Function

### 2.1. Physiological Function

#### 2.1.1. Synaptic Role of ASIC2

ASIC1a has been widely explored in synaptic plasticity due to its calcium permeability and membrane depolarization [41,42,43,44,45,46,47]. Relating to ASIC2, ASIC1a forms multimeric complexes with ASIC2 subunits and has its expression promoted by ASIC2 [9,10]. These interactions suggest that ASIC2 may also serve a modulatory role in synaptic plasticity [17,41]. Expanding on this idea, ASIC2 presence is important in maintaining synapses and dendritic spines in normal physiology [48]. The immunostaining of mice brains showed that ASIC2 in the CNS is found in the synapses of neurons and is found at lower concentrations along axons [48]. ASIC2 is also required for maintaining synapse and dendritic spine density [48]. It was shown that ASIC2a facilitated the trafficking of ASIC1a to dendritic spines, and disruption of the ASIC2 gene resulted in decreases in both synapse and dendritic spine densities, likely due to reduced ASIC1a expression [48].

**Figure 2:**
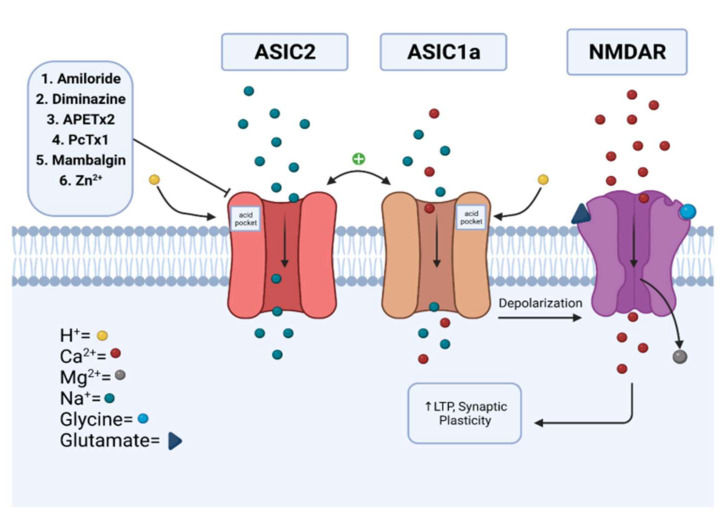
Synaptic role of ASIC2 in promoting LTP and synaptic plasticity. ASIC2 both upregulates and forms complexes with ASIC1a and plays a role in membrane depolarization and NMDAR activation. Also depicted are the molecules reviewed in this paper which modulate ASIC2 channels. Listed are modulators of 1–6; each of these modulate ASIC2 channels through various mechanisms and lead to altered sodium and/or calcium influx. The labeled protons excite the subunit complexes. NMDAR: *N*-methyl-*D*-aspartate receptor. LTP: long-term potentiation. Adapted from “Transporters” by BioRender.com (2021). Retrieved from https://app.biorender.com/biorender-templates, accessed on 1 December 2021.

Synaptic plasticity is an important process in the body which mediates emotions, thought processes, and behavior by increasing the number of neurotransmitter receptors and neurotransmitter release [49]. ASICs are thought to have important roles in enhancing the formation of synaptic plasticity, particularly in the hippocampus [46]. The most studied models of synaptic plasticity are long-term potentiation (LTP) and long-term depreciation (LTD), whose induction involves the α-amino-3-hydroxy-5-methyl-4-isoxazolepropionic acid receptor (AMPAR) and *N*-methyl-*D*-aspartate receptor (NMDAR) at synaptic membrane sites [49,50]. These are seen throughout the brain, especially in the CA1 region of the hippocampus through NMDARs and the anterior cingulate cortex [44,46]. Although the NMDAR functions through glutamate and glycine neurotransmitter binding, it is known that protons can also function as a neurotransmitter [47]. Specifically, the proton activation of ASIC1a leads to membrane depolarization and could assist in removing the Mg^2+^ ion block of NMDARs which, in turn, increases the probability of forming LTP and establishing synaptic plasticity [47]. ASIC2 increases the membrane trafficking of ASIC1a and forms homotrimers and heterotrimer complexes with ASIC1a; therefore, the presence of ASIC2 is important to synaptic plasticity through the upregulation and modulation of ASIC1a (Figure 2) [48]. Further studies showed a decrease in the AMPAR/NMDAR ratio (indicative of LTD) in the brains of ASIC1a knockout (KO) mice as compared with wild-type (WT) mice [46].

Recent research has also shown that ASIC1a and ASIC2a are linked to fear-related behaviors [51,52]. Similarly to ASIC1a, ASIC2 was seen to be highly expressed in the fear-related areas of the brain, such as the basolateral and central nucleus of the amygdala, and bed nucleus of the stria terminalis [51,52]. ASIC2 is also present in the CA2 region of the hippocampus, which responds to threat stimuli and modulates learning and memory in stress-related situations [51]. Researchers used ASIC1, ASIC2, and ASIC1/2 KO mice to test the role of ASICs in response to aversive stimuli and associated motor responses such as the duration of freezing behavior following such stimuli [51]. Results confirmed that all three categories of ASIC null mice exhibited less freezing behavior in a series of fear-conditioning experiments [51]. Thus, similarly to other ASIC subtypes, ASIC2 is implicated in normal fear physiology.

#### 2.1.2. Mechanosensation of ASIC2

Early studies discovered that ASICs were found in *Caenorhabditis elegans* and functioned in transducing mechanical force in touch sensation to neuronal signals [53,54]. Since then, more studies have elucidated the mechanosensory functions of ASICs, including ASIC2. It is now understood that ASIC2 and others of the DEG/ENaC protein family are localized in cutaneous and DRG sensory afferents in humans and rodents [53,54,55,56,57]. Specifically, immunohistochemistry shows the presence of ASIC2 in Meissner corpuscles, Pacinian corpuscles, Merkel discs, and nerve endings surrounding hair follicles in hairy skin [55,56,57]. ASIC2 is important in physiological mechanosensation and muscle mechanotransduction [55,58]. Assays with immunohistochemistry, immunoblot, and patch clamp recording collectively demonstrate that ASIC2, similarly to other members of the DEG/ENaC family, is among the primary mechanotransducers in muscular spindle fibers [55,58].

Although it is known that an increase in acid concentration can induce ASIC2 inward currents, ASIC2 mechanosensation has been speculated to be proton-independent due to a failure in pH drop to stimulate ASICs in peripheral DRG neurons [53,54]. How exactly are ASIC2 and other DEG/ENaC members activated through touch? A likely mechanism proposed by Welsh’s group is that mechanical forces activate ASICs due to the channels being bound to the extracellular matrix and/or the intracellular cytoskeleton [53]. This notion is supported by the fact that ASIC2 is extensively expressed within cutaneous structures [55,56,57]. In contrast, acid is not arbitrary in ASIC-related mechanosensation. Recent studies demonstrate that pH is a necessary component, along with shear force, in inducing ASIC activity [59]. Although shear force (provided by fluid inflow perfusion) alone failed to induce ASIC2a activation at pH 7.4, a combination of increased shear force, decreased pH (pH between 6.0 and 4.0), and pre-activation of ASICs with non-proton ligands such as MitTx and GMQ provoked inward currents, with the latter occurring at pH 7.4 [59].

Previously, ASIC2 was considered to be less likely to be required in normal hearing physiology, despite its strong prevalence in spiral ganglia in cochlea [60,61]. Similar to the combination of stimuli in a shear force test of ASIC2 mechanosensation, assays of ASICs in the cochlea involved pH manipulation alongside square and sinusoidal currents to observe ASIC2 behavior [62]. Unsurprisingly, more acidic pH solutions elicit stronger spiral ganglion neuron (SGN) depolarization, which led to the enhanced inhibition of action potentials from these neurons [62]. Despite these findings, studies with ASIC2 KO mice demonstrate that the lack of this channel protein did not cause substantial changes in hearing sensitivity, but it increased resistance to temporary noise-induced threshold shifts [60,61,62]. Conflicting findings regarding ASIC2 in hearing mechanotransduction make pinpointing its role in normal physiology challenging, especially when mechanosensory research on specific ASIC2 subunits is lacking. Future examination of individual ASIC2a and ASIC2b may help clarify the subunit-specific roles of these ASICs in cochlear mechanosensation. In summary, although details of ASIC2 participation in mechanosensation remain to be fully elucidated, this channel certainly plays an integral role in mechanotransduction.

ASIC2 has also been implicated in the autonomic baroreception and myogenic regulation of renal blood flow [63,64]. ASICs were expressed in the nodose ganglia [63]. Nodose ganglia baroreceptors function to lower heart rate in response to elevated blood pressure [65]. ASIC2-null mice were both hypertensive and tachycardic, even at a lower level of activity as compared with WT mice; ASIC2-null mice also showed a decreased baroreceptor reflex gain [63]. In renal vasculature, ASIC2 exerts protective myogenic vasoconstriction to correct changes in renal perfusion pressure [64]. Additionally, the myogenic regulation of renal vasculature was attenuated in ASIC2^+/−^ and ASIC2^−/−^ mice along with an elevation of TGF-β, a marker of renal fibrosis [64]. Taken together, these findings demonstrate that a loss of ASIC2 results in hypertension and pressure-related renal injury [63,64]. Thus, ASIC2 is necessary for the proper maintenance of both blood pressure and renal perfusion [15].

### 2.2. Pathology of ASIC2

**Figure 3:**
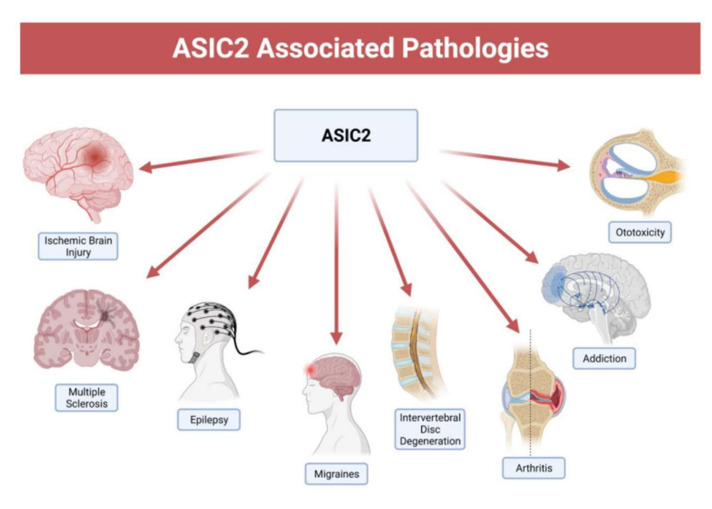
ASIC2-associated pathologies. ASIC2 has been implicated in a variety of diseases such as ischemia, multiple sclerosis, epilepsy, migraines, intervertebral disc degeneration, arthritis, addiction, and aminoglycoside-induced hearing loss. Although the relationship between ASIC2 and some of these diseases can be more established with future studies, there is promise that future medical interventions targeting ASIC2 can be beneficial. Adapted from “Complications of Hypertension” by BioRender.com (2021). Retrieved from https://app.biorender.com/biorender-templates, accessed on 1 December 2021.

As mentioned above, ASICs are implicated in neurological, psychological, and cardiovascular diseases. In this section, we will focus on the contribution of ASIC2 to the pathogenesis and symptomatology of these diseases (see Figure 3).

#### 2.2.1. Ischemic Brain Injury

Brain acidosis is one important contributor to cell injury in ischemic events [66,67]. Due to the sensitivity of ASICs to acidic environments, ASIC1a likely mediates neuronal injury induced by ischemia [17,68,69,70]. Most of the investigations into ASICs during ischemia have revolved around ASIC1a, but some have examined the contribution of ASIC2 [17]. A study from Dr. Simon’s group found that surviving neurons from global ischemic events in an MCAO model had an increased level of ASIC2a expression, suggesting a potential protective role of ASIC2a [71]. Another group found that the expression of ASIC2a was significantly increased after ischemia/reperfusion, whereas the ASIC1a and ASIC2b levels remained unchanged [70]. Our studies found that ASIC2 deletion actually reduced acid-activated currents and intracellular calcium increases in hippocampal neurons [17,37,48]. In a study aimed to examine the brain-region-specific role of ASIC2, we found that ASIC2 deletion had no impact on acid-mediated responses in the cerebellum. Notably, ASIC2 deficit exerted a significant influence in the hippocampus, cortex, and striatum [17]. Specifically, the deletion of ASIC2 led to a reduction in acid-activated currents in the neurons of these regions [17].

Further inquiry into the mechanism behind these observed changes in ASIC2 and acid-mediated responses showed that ASIC2 deletion impacted ASIC1 expression in general and at the neuronal surface [17]. In the cerebellum, striatum, hippocampus, and cortex, ASIC1a expression was reduced in ASIC2^−/−^ mice. Intriguingly, the surface expression of ASIC1a in the cerebellum was unchanged, leading to an increase in the surface: total ratio of ASIC1a. In the striatum, surface ASIC1a expression was reduced and the surface: total ratio was decreased. In the hippocampus and cortex, surface ASIC1a was also decreased, as in the striatum, but the surface: total ratio of ASIC1a was not changed [17].

The regional differences caused by ASIC2 deletion may result from different levels of expression of ASIC2a and ASIC2b in these regions [17]. One study found that ASIC2b is the dominant ASIC2 subunit in the cerebellum, whereas ASIC2a is the predominant ASIC2 subunit in the striatum. There is an equal balance in ASIC2a versus 2b expression in the hippocampus and cortex [10]. Further prompting led to experiments investigating the mechanism by which ASIC2 influences ASIC1a expression. Evidence indicates that ASIC2 assists the maturation of *N*-linked glycans of ASIC1a, in which ASIC2a is more efficient than ASIC2b, and thus modifies ASIC1a expression [17]. Together, the brain regional differences and the mechanism underlying the ASIC2-mediated modulation of ASIC1a expression emphasize the complexity of ASICs and the specific influence of ASIC2 variants over the damage derived from ischemic events.

#### 2.2.2. Multiple Sclerosis

Nearly one million adults in the United States live with multiple sclerosis (MS) [72]. MS is an autoimmune disease which leads to the demyelination of axons in both the brain and spinal cord [72]. Recent investigations into the pathophysiology of MS revealed that Ca^2+^ and Na^+^ concentrations have an influence on the extent of damage to myelin [73,74]. ASIC1 activation plays a crucial role in the accumulation of these ions [75,76]. In fact, blocking ASIC1 via PcTx1 provides a neuroprotective effect against axonal degeneration [73,74]. With drops in pH from roughly 7.4 to 6.5 in CNS inflammatory lesions, ASIC1 homotrimers can be activated. However, this degree of pH drop is insufficient to activate ASIC2 variants [76]. Although this seems to suggest a possible absence of ASIC2 activation during MS, genetic studies identified a significant association between ASIC2 and polymorphisms in MS [76,77].

These findings motivated further experiments, which examined the influence of ASIC2 in mice with experimental autoimmune encephalomyelitis (EAE), a mouse model of MS [78]. In one experiment, with the induction of EAE in WT mice, ASIC1^−/−^, and ASIC2^−/−^ showed a slowed onset of clinical changes in both ASIC1^−/−^ and ASIC2^−/−^ mice [78]. However, immunohistochemical analysis of the spinal cord of EAE mice showed that ASIC2^−/−^ mice exhibited an increase in CD4^+^ mononuclear cells compared with WT mice, and no changes in MHC-II or CD8^+^ [78]. These findings together suggest that ASIC2 plays a role in the pathology of MS, although it may act through modifying ASIC1.

#### 2.2.3. Epilepsy

The involvement of ASICs, including ASIC2, is observed in neuronal excitability, especially in relation to imbalances in neuronal excitation seen in seizure and epilepsy [79,80,81]. Amiloride has been shown to reduce generalized seizures induced by either pentylenetetrazole or electrical stimulation [18,82]. Following pilocarpine administration, amiloride delayed the occurrence of status epilepticus and the onset of a first seizure episode [18,82]. In the hippocampus, the inhibition of ASICs reduced epileptic discharges in a low-Mg^+^ model [83]. Even at the genetic level, ASIC1a expression is related to seizure and epilepsy [79,84]. One study found that those with temporal lobe epilepsy had significant allelic and genotypic association with a specific ASC1a variant allele, in addition to a significant association found in their haplotype analysis [84].

One of the strongest collections of evidence suggesting ASIC2’s specific involvement is how changes in ASIC2a expression affect the intrinsic excitability of pyramidal CA1 neurons [18]. Neurons with an overexpression of ASIC2a fired more frequently than control neurons at all current injections over 150 pA [18]. In line with the conclusion that ASIC2a exerts an impact on neuronal excitability, neurons with a lower level of ASIC2a expression fired significantly fewer action potentials than controls at all current injections above 150 pA [18]. In addition, through in vivo investigations looking at the seizure behavior of negative control rats versus rats transfected with ASIC2a-expressing adeno-associated viruses, it was found that ASIC2a overexpression significantly accelerated the onset of the first seizure episode, reaching Racine stage IV (rearing with forelimb clonus) and increased the occurrence of status epilepticus episodes that reached Racine stage IV [18]. ASIC2a overexpression also increased susceptibility to stimulated seizures [85]. Together, these lines of evidence clearly support that ASIC2a possesses the ability to influence epilepsy.

#### 2.2.4. Migraines

Migraines and associated pain have debilitating effects on the individuals who experience them. Research into their treatment has not led to a universally effective treatment or complete description of the pathophysiology [19]. Investigations into the influence of ASICs on migraines have revealed some distinct connections. In a rodent model, ASIC1 and ASIC3 have been shown in migraines [86,87]. In migraines, meningeal pH is reduced, which provides a reasonable hypothesis for the mechanism behind ASIC2 subunits being involved in pain processing [19]. Intriguingly, if low pH is applied to the dura of awake animals, these animals displayed behaviors that mirrored those shown in headaches [88]. Even more convincingly, this effect, triggered by a small drop in pH, can be blocked by non-selective ASIC blocker amiloride, suggesting that ASICs might play the most significant role in this process [88]. In summary, there is no direct evidence to support ASIC2 responsible for migraines. Future studies may investigate this in an ASIC2 KO mouse migraine model to see whether it is involved.

#### 2.2.5. Intervertebral Disc Degeneration

Another degenerative disorder in which ASICs are suggested to have a role is intervertebral disc degeneration (IVDD) [76]. This is the chronic destruction of the extracellular matrix, which leads to lower back pain [89,90]. Part of IVDD involves anaerobic metabolism on the part of the disc cells, leading to increased levels of lactic acid, which are further enhanced by the influence of cytokines [91,92]. Of course, this acidic environment is not conducive to gene expression, proliferation, or the viability of disc cells [93]. Due to this pathophysiology, investigation into the expression of ASICs in these areas found an upregulation of ASIC1, ASIC2, and ASIC3 in the nucleus pulposus [20]. Even more captivatingly, an investigation found that the acid-induced elevation of Ca^2+^ via ASIC1a was directly involved in endplate chondrocyte apoptosis, and the subsequent inhibition of ASIC1a via PcTx1 led to reductions in acid-induced apoptosis and Ca^2+^ levels [94]. These findings again suggest a pathological role of ASICs, with ASIC2 potentially mediating the damage via its modulation of ASIC1a proteins.

#### 2.2.6. Arthritis

Following these same lines, inquiry into ASIC involvement in arthritis found that amiloride reduced the Ca^2+^ increase seen in articular chondrocytes exposed to extracellular pH 6.0, and attenuated acid-induced articular chondrocyte injury [95]. This is likely because matrix turnover is influenced by changes in extracellular acidosis [96]. Mechanistically, another study found that interleukin-6 enhanced acid-induced apoptosis via the upregulation of ASIC1a expression, supporting these arthritis findings and providing a potential piece of the pathophysiological puzzle of ASIC involvement [21]. Similar to the neurodegenerative evidence, investigations into IVDD and arthritis have not specified ASIC2 as having a bigger role than ASIC1a. However, the upregulation in the nucleus pulposus for IVDD and the potential modulation of ASIC1a by ASIC2 in the arthritic process highlight the role of ASIC2 in these pathologies. Future studies should determine whether ASIC2 is directly involved in arthritis and IVDD.

#### 2.2.7. Addiction

Addiction is defined as a “chronic, relapsing disorder characterized by compulsive drug seeking, continued use despite harmful consequences, and long-lasting changes in the brain” [97,98,99]. Many neurotransmitter systems have been implicated in the pathology of this disease, with a majority of the attention surrounding dopaminergic and glutamatergic transmission in the nucleus accumbens (NAc) [100]. Interestingly, acid-sensing receptors have been documented as relevant mediators in the addiction pathway [101,102,103,104]. When ASIC1a was deleted in mice, cocaine-conditioned preference was increased, and this can be reversed by the rescue of ASIC1a in the NAc [22,102]. Additionally, increased ASIC1a in the NAc led to decreased cocaine self-administration and a rightward shift in the dose–response curve [22,102]. Even after 14 days of abstinence following 5 days of experimenter-administered cocaine, ASIC1 expression was elevated in the NAc [103].

These findings substantiate the role of ASIC1a in addiction, although they could not specify the role of the ASIC2 subunit beyond their modification of ASIC1a. Further examinations by Kreple et al. found that in the presence of ASIC2 activity, postsynaptic transmission in the NAc was mediated by ASIC1a/2a heteromeric channels rather than ASIC1a homomers [22]. This importantly links the ASIC1a influence with the ASIC2 subunit, not as a standalone. Our studies have shown that amphetamine increased the surface expression of ASIC2 in the rat medial prefrontal cortex (mPFC), as opposed to an insignificant change in ASIC1 expression in this region [23]. These changes were reversed in the NAc, with an increase in the surface and intracellular expression of ASIC1 and no change in ASIC2 expression [23]. In response to a cocaine challenge, ASIC1a KO mice retained their behavioral sensitization [23]. Interestingly, ASIC2 KO mice displayed significantly less sensitization to their cocaine challenge as compared with WT and ASIC1a KO mice [104]. These findings suggest that although ASIC1a and ASIC2a are related in their influence on addiction, they may play different roles in drug addiction, at least in a cocaine sensitization paradigm [23]. In all, ASICs contribute to addiction and ASIC2a plays a significant role in this event.

#### 2.2.8. Aminoglycoside-Induced Hearing Loss

One of the unfortunate potential side effects of aminoglycoside antibiotics is ototoxicity [105]. Previous research has identified the mechanism of injury as the disruption of protein synthesis in mitochondria, the formation of free radicals, and overactivation of NMDARs [105,106]. Interestingly, one study found that these antibiotics increased the ASIC currents in DRG neurons [107]. Additionally, the same molecules directly increased ASIC currents in spiral ganglion neurons (SGNs), which could contribute further to the observed ototoxicity [62]. Further examination into the ASIC role in hearing found that ASICs in SGNs were activated by the mechanical activation of hair cells, leading to proton release or by a Na^+^/H^+^ exchanger [62,108]. Immunohistochemical findings showed the expression of both ASIC2a and ASIC2b in SGNs, solidifying their specific importance in this pathology [62]. Other research shows protons being used as messengers of intercellular communication in the auditory and vestibular systems of multiple species [109]. Further investigations may reveal potential treatment options targeted at inhibiting these ASIC2 subunits.

## 3. Modulation

The pharmacological modulation of ASIC2 becomes increasingly important for studying the physiology and pathology of ASIC2. A table including chemical and 3D (from protein data bank) structures of respective modulators is included for the readers’ reference (Table 1). We next briefly discuss several major inhibitors and modulators of ASIC2 (also see Figure 2).

### 3.1. Amiloride

Amiloride is a non-selective blocker of ASICs and is a potential therapeutic option for pathologies associated with ASIC activation [7,11,110]. Amiloride is a potassium-sparing diuretic medication, but in the case of ASICs, it prevents acid-induced calcium and sodium influx associated with nociception [8,11,110]. This is especially useful in the case of myocardial ischemia, where ASIC2a/3 heterotrimers are seen to have pH-evoked currents in cardiac DRG neurons for afferent pain signaling [39]. Amiloride was shown to be effective at pH levels of 6.5, reducing acid-evoked pain sensation down to the pH 7.4 control level [110]. However, at a pH value of 5.0, these analgesic properties were reduced [110]. These results suggest that amiloride is a possible therapeutic option for attenuating acid-evoked pain at pH levels over 6.0 [11,110].

### 3.2. Diminazene

Diminazene is a potent, non-selective inhibitor of ASICs, but shows varying levels of inhibition within the subtypes of ASIC1, ASIC2, and ASIC3 [111]. All ASIC homomers other than ASIC2a were more potently inhibited by diminazene [111]. Further testing with pH stimulus solutions showed that diminazene was more effective in ASIC inhibition at lower pH levels [111]. Results from these solutions, as well as patch clamp recording, indicate that diminazene primarily functions as an open-channel blocker (when ASICs are activated), and its inhibition of ASICs is pH-dependent [111,112]. Following this idea, the currently proposed order of diminazene inhibitory potency is ASIC1b > 3 > 2a ≥ 1a [110,111]. Another effect of diminazene in the inhibition of ASICs is anti-hyperalgesia, which has been shown to be similar to the effects of morphine in rat models [111]. The diminazene-induced high-affinity inhibition of ASICs could make it useful in clinical settings, especially in ischemia or inflammation with pH drops [110].

### 3.3. APETx2

APETx2 is an animal toxin derived from *Anthopleura elegantissima*. Similar to other sea anemone toxins, APETx2 is thought to be a prospective analgesic intervention [113,114,115]. APETx2 is mostly associated with decreasing pain through the selective inhibition of ASIC3 homomers, as shown in chronic pain models in rodents [115]. However, its exact role in other ASIC subunits is not well understood. One study found that in addition to ASIC3, APETx2 demonstrates an inhibitory effect on other ASIC subunits such as ASIC1b and ASIC2a at certain concentrations [111]. Specifically, ASIC3 is believed to form heteromers with ASIC2a, and such heteromers are sensitive to APETx2 inhibition in DRG neurons [116]. APETx2 also shows anti-hyperalgesia effects, similar to morphine, but showing no stable plateau in dose-dependent pain reduction at higher dosages [111]. In summary, the present data support the idea that APETx2 acts on ASIC2-containing channels and is a substantial inhibitor of pain in chronic inflammatory conditions [116].

### 3.4. Psalmotoxin (PcTx1)

PcTx1 is a peptide derived from venom of the South American tarantula *Psalmopoeus cambridgei* [116,117]. PcTx1 is a potent inhibitor of homomeric ASIC1a channels, and heteromeric ASIC1a/2b and ASIC1a/2a channels [29,118]. Mechanisms underlying the effect of PcTx1 involve its interactions with the thumb, finger, and palm domains of ASICs, which block their arrangements in the desensitized state [116,117]. Interestingly, PcTx1 has antiproliferative properties in human lung adenocarcinoma A549 cells, which express ASIC1, ASIC2, and ASIC3 [119]. Although most studies about the neuroprotective effect of PcTx1 primarily target ASIC1a inhibition, PcTx1 is a potent inhibitor of both homomers and heteromers made of both ASIC1 and ASIC2 subunits [29,118,119]. Evidence points to this animal-toxin-derived peptide having beneficial therapeutic potential, although more research is needed to further understand precisely how PcTx1 modulates ASIC2a and ASIC2b channels.

### 3.5. Mambalgins

Other animal toxins that have exhibited inhibitory properties of ASICs are mambalgins [120,121,122,123,124,125]. As a toxin derived from the venom of *Dendroaspis polylepis* [120], mambalgins are classified into three subtypes (mambalgin-1, -2, -3) which function similarly to inhibit all ASIC subunit combinations such as heteromeric ASIC1a/2a but not individual ASIC2a [120]. Mambalgins bind to the closed-state of ASICs, which shifts the pH-dependent activation of the channel to a more acidic state. This ultimately leads to the decreased excitability of ASICs in an acidic environment [121]. ASIC2-containing heteromers play a role in nociception; therefore, the inhibition of ASIC1a/2a by mambalgins is believed to function in central analgesia [120,124]. Consistent with this idea, mambalgin injections into the CNS evoke opioid-independent analgesic pathways involving ASIC1a and ASIC2a [124]. Thus, mambalgin is a naturally occurring peptide that shows potential therapeutic value in pain reduction via a mechanism involving ASIC2.

### 3.6. Zinc

As an endogenous biomolecule, Zn^2+^ potentiates the acid activation of homomeric and heteromeric ASIC2a-containing channels (i.e., ASIC2a, ASIC1a/2a, ASIC2a/3) in micromolar ranges, but not that of homomeric ASIC1a and ASIC3 [126]. Our own studies demonstrated that the modulation of zinc ions on ASICs can be described as a dual effect [127]. Specifically, Zn^2+^ inhibits ASIC1a and heteromeric ASIC2a/1a at nanomolar concentrations, although potentiates ASIC2a and heteromeric ASIC2a/1a at micromolar concentrations [127]. Inhibition of ASICs by Zn^2+^ occurs on the extracellular side. This is clarified by the application of membrane-impermeable and -permeable Zn^2+^ chelators [127]. Potentiation with higher Zn^2+^ concentrations is supported by results from a study testing the effects of 100 μm Zn^2+^ on ASIC2a-mediated currents [127]. Specifically, ASIC2a-containing channels exhibited an increased current after the addition of Zn^2+^ at pH 6.5 [127]. ASIC2a has activation pH levels below 5.5, which indicates that Zn^2+^ potentiation most likely occurs to ASIC2 heteromers such as ASIC2a/1a [127]. Interestingly, ASIC2a/1a-mediated currents may induce membrane depolarization, which allows Ca^2+^ influx through voltage-gated calcium channels and NMDARs [41]. Thus, the inhibition of ASIC2a/1a by nanomolar concentrations of Zn^2+^ may have potential to prevent neuronal excitotoxicity under pathological conditions [41,127].

## 4. Perspective

ASIC2 subunits have been in the shadow of other more densely populated ASIC1 members, but recent research has established significant and unique roles of these acid-sensing proteins in physiology and diseases [28]. They sense acidotic changes in ischemic environments, manage cardiac afferent neuron activity in cardiac reflexes, facilitate LTP, propagate neurological fear responses, and influence autonomic baroreceptor activity in the regulation of renal and cardiac blood flow. They are implicated in the pathologies of ischemia, multiple sclerosis, epilepsy, migraines, intervertebral disc degeneration, arthritis, drug addiction, and aminoglycoside-induced hearing loss (Table 2). In addition, another set of pathologies may prove to be relevant to ASIC2 in the future.

Three neurodegenerative diseases are suggested to have ASICs involved in their pathology, including Parkinson’s disease (PD), Huntington’s disease (HD), and Alzheimer’s disease (AD) [26,76,128]. PD is a neurodegenerative disease that impacts the dopaminergic neurons of the midbrain, which leads to motor dysfunction [129]. Studies using inhibitors of ASICs, such as amiloride and PcTx1, have shown a preservation of dopaminergic neurons in the substantia nigra and reduced loss of dopaminergic transmission in the striatum, respectively [130]. Although one study with ASIC1a KO mice found no alteration in neurodegeneration in a subacute MPTP model of PD [131], a more recent study using paeoniflorin (the major active component of the total glycoside of paeony for treatments of pain and inflammation) rather than amiloride to inhibit ASIC1a subunits found improvements in behavioral symptoms, delayed dopamine neuron loss, and attenuation in the reduction in dopamine transmission [24]. Specific mechanisms include ASIC signaling acting as a factor in the parkin-mediated monoubiquitylation of proteins interacting with C kinase 1, which contributes to the neuronal degeneration seen in PD [132].

HD is a rare neurodegenerative disorder which causes progressive cognitive decline, personality change, and characteristic movement issues [133]. Interestingly, a commonly reported feature of its pathophysiology is metabolic impairment, leading to the accumulation of lactic acid in the CNS [134,135]. Due to these findings, one group investigated the use of an amiloride derivative, benzamil, and found that it had therapeutic effects in the R6/2 animal model of HD [136]. Mechanistically, ASICs influence the aggregation of htt-polyQ, suggesting that the blockage of their activity could prove a therapeutic for those with HD [76,136]. These findings for both PD and HD show that ASICs are involved in the pathology of neurodegenerative diseases [76].

AD and its mechanisms have been the focus of scientific investigation for decades. Some suggest that an acidic environment found in AD patients’ brains is related to ASIC function [25]. ASIC1a, in the presence of Aβ plaques and an agonist for group I metabotropic glutamate receptors, led to an increase in the excitability of hippocampal neurons, a set of neurons central to the pathology of AD [25]. The well-known AD medication memantine has been shown to inhibit ASIC1a, suggesting that some of its benefits may be derived from its impact on ASICs, in addition to its NMDAR inhibition [137]. However, in the light of some contradictory evidence and no distinct data specifying the ASIC2 contribution, there is a need to further investigate the unique role of these channels in these neurodegenerative diseases.

Another uncertain area pertinent to our understanding of acid-sensing channels involves other proteins that are activated by protons. One example is the proton-activated chloride channel (PAC), also known as the acid-sensitive outward rectifying anion channel (ASOR) [138]. These channels are formed by TMEM206 proteins, which are expressed at the plasma membrane and are suspected to have orthologs in all vertebrates [138]. One study found that zebrafish with no PAC had attenuated brain damage after ischemic stroke [139]. Outside of PAC, a different class of receptors has also been identified, which is called the G-protein-coupled proton-sensing receptor (GPR), including GPR4, GPR68, and GPR65 [140]. It was found that abolishing GPR4 in the mouse retrotrapezoid nucleus (RTN) led to increased apnea frequency and blunted ventilatory responses to CO_2_ [141]. The reintroduction of these receptors restored the ventilatory phenotype and CO_2_-dependent RTN neuronal activation [141]. A recent investigation found that GPR68 is protective against neuronal injury under ischemic and acidotic conditions [142]. A separate investigation found that in GPR68 KO mice, there was reduced LTP, and that those mice exhibited reduced avoidance to a dark chamber [143]. Acid-sensing receptors are widely expressed in immune cells as well, suggesting potential roles beyond our current understanding [144]. A novel mechanism for CO_2_-evoked fear was suggested to involve the microglial acid-sensing GPCR in the subfornical region [144]. In total, these contemporary investigations lay an important foundation for future investigations into all of the proteins that mediate physiological and pathological responses to acidic changes.

However, it is important to acknowledge that the majority of the evidence listed in this paper is either from in vitro or in animal models. Future experiments must examine the role of these proteins in humans. Additionally, much of ASIC2’s influence is purported to be via its modulation of the surface expression of ASIC1a. Although this still supports an active role for ASIC2 subunits, it leaves questions regarding the specific mechanism of these cellular modifications. Outside of the lack of human data and questions of ASIC2 causality, there are insufficiently trialed therapeutic interventions targeting ASIC2 subunits specifically. Medications such as memantine which treat AD have been found to modify ASICs, but that is not their primary mechanism of action.

Future pharmacological targets could include ASIC2 inhibition in the treatment of addiction, supported by the evidence that ASIC2 KO mice were less sensitized to a cocaine challenge than WT and ASIC1a KO mice [104]. Additionally, ASIC2a inhibition for the purpose of epilepsy treatment could be meaningful, based on the findings that ASIC2a overexpression increased the susceptibility to stimulated seizures and accelerated the onset of status epilepticus [18,85]. In the emergent setting, applications of ASIC2 inhibition may be beneficial for patients with ischemic events due to the results showing that ASIC2 deletion reduced acid-mediated responses in the hippocampus, cortex, and striatum [17]. For the incessant problem of migraines, drugs targeting ASIC2 subunits in ASIC1a/2a heterotrimers may well reduce the pain experienced by patients [125]. In neurodegenerative diseases in which there are issues of inflammation such as IVDD and arthritis and antibiotic-induced hearing loss, more evidence identifying ASIC2’s relevant function is needed before interventions can realistically be endeavored.

There is a wealth of promise in the potential benefit of medical interventions attacking these acid-sensing channels. Investigations into the effects of pharmacological intervention targeting ASIC2 subunits *in vivo*, eventually progressing to the point of human clinical trials, will reveal the merit of the accumulated evidence. In summary, this review leaves no question that these channels, at times, perpetuate damage, and thus are reasonable targets for treatment. Continued exploration will shed light on the efficacy of these theoretical treatments and may lead to a development in the way patients are treated.

## Figures and Tables

**Table 1:** ASIC2 Modulators.

Modulators	Chemical and 3D Structures
**Amiloride**	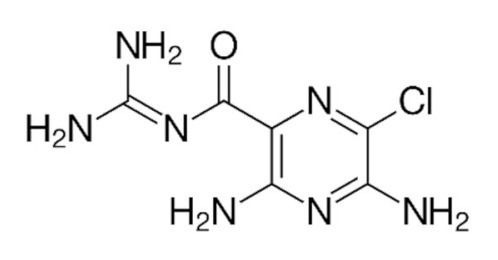
**Diminazene**	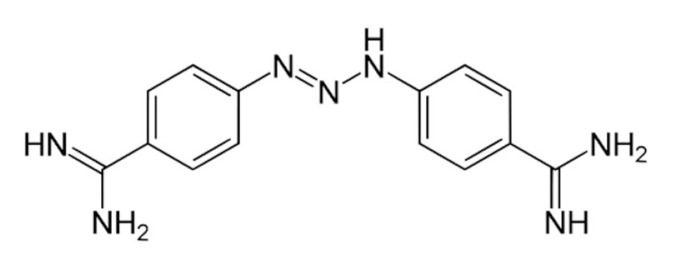
**APETx2** **(PDB 2MUB)**	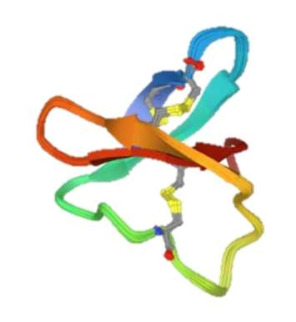
**PcTx1** **(PDB 2KNI)**	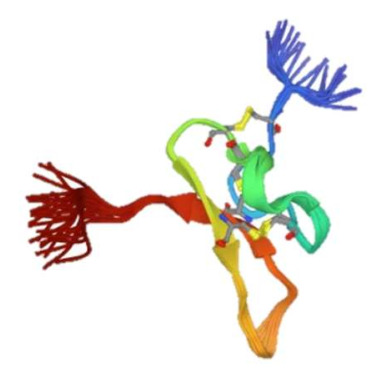
**Mambalgin 2** **(PDB 2MFA)**	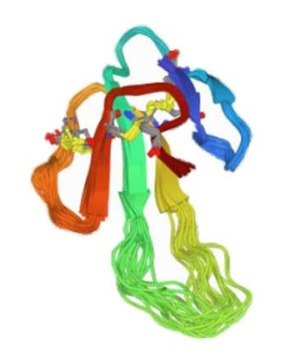
**zinc**	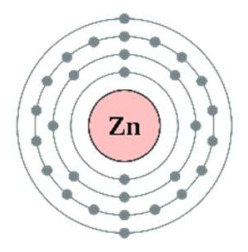

Chemical and 3D structures of modulators of ASIC2—Amiloride, Diminazene, APETx2 (PDB 2MUB) from sea anemone venom, PcTx1 (PDB 2KNI) from spider venom, Mambalgin 2 (PDB 2MFA) from snake venom, and zinc. PDB: protein data bank.

**Table 2:** ASIC2 Pathologies.

Pathologies	ASIC2 Association from Findings
**Ischemic Brain Injury**	Increased ASIC2a in surviving neurons from global ischemic events, with unchanged ASIC1a and ASIC2b [70,71] Reduces acid-activated currents primarily in hippocampus, cortex, and striatum [17] ASIC2a and ASIC2b assists ASIC1a expression which is also implicated in acid-evoked ischemic injury [17]
**Multiple Sclerosis**	Knockout in mice slows clinical pathogenesis [78] Inhibition of ASIC1a in rats, mediated by ASIC2, prevents axonal/myelin degeneration [78] Knockout mice had increased CD4^+^ cells compared to WT mice [78] Knockout mice had no change in MHC-II or CD8^+^ cells [78]
**Epilepsy**	Observed in neuronal excitability imbalances [18,81] Inhibition delays status epilepticus and first seizure episode onset in rodents [18,81] Overexpression increases neuronal excitability and accerlates seizure onset in rodents [85]
**Migraines**	Reduced acute and inflammatory pain with ASIC1a-2a heteroreceptor inhibition in rodents [27] Involved in migraine-associated pain processing rodents [86,87,88]
**Intervertebral Disc Degeneration**	Upregulation of ASIC2 in nucleus pulposus [20] Inhibition of channels mediated by ASIC2 led to reduced acid-induced apoptosis and Ca^2+^ levels [94]
**Arthritis**	Inhibition reduced Ca^2+^ increase seen in articular chondrocyte injury [21,95]
**Addiction**	Significant role of ASIC2a for addiction inhibition [102,104] Mediation of ASIC1a implicated in rodent cocaine addiction pathways [101,102,103] Mouse model demonstrated reduced sensitizaion in response to cocaine administration [104]

Data were drawn from numerous studies performed on both rodent and human models to determine ASIC2 function in a variety of disease as shown. ASIC2 is seen to have both direct association with these diseases as well as being a primary mediator of other ASICs which are involved in the respective pathologies.

## Data Availability

Not applicable.

## Abbreviations

AD	Alzheimer’s disease
AMPAR	*α*-amino-3-hydroxy-5-methylisoxazole-4-propionic acid receptor
ASICs	acid-sensing ion channels
ASOR	acid-sensitive outward rectifying anion channel
CNS	central nervous system
DEG/ENaC	degenerin/epithelial sodium channel
DRG	dorsal root ganglion
EAE	experimental autoimmune encephalitis
GPCR	G-protein-coupled receptor
GPR	G-protein-coupled proton-sensing receptor
HD	Huntingdon’s disease
IVDD	intervertebral disc degeneration
KO	Knock-out
LTD	long-term depression
LTP	long-term potentiation
MCAO	middle cerebral artery occlusion
mPFC	medial prefrontal cortex
MS	multiple sclerosis
NAc	nucleus accumbens
NMDAR	*N*-methyl-D-aspartate receptor
PAC	proton-activated chloride channel
PcTx1	psalmotoxin-1
PD	Parkinson’s disease
PDB	protein data bank
RTN	retrotrapezoid nucleus
SGNs	spiral ganglion neurons
TM	transmembrane domain
WT	wild-type
